# Calcium‐sensing receptor regulates intestinal dipeptide absorption via Ca^2+^ signaling and IK_Ca_ activation

**DOI:** 10.14814/phy2.14337

**Published:** 2020-01-21

**Authors:** Jingyu Xu, Andre Zeug, Brigitte Riederer, Sunil Yeruva, Oliver Griesbeck, Hannelore Daniel, Biguang Tuo, Evgeni Ponimaskin, Hui Dong, Ursula Seidler

**Affiliations:** ^1^ Department of Gastroenterology, Hepatology and Endocrinology Hannover Medical School Hannover Germany; ^2^ Research Gastroenterology Affiliated Hospital of Zunyi Medical University Zunyi China; ^3^ Cellular Neurophysiology Hannover Medical School Hannover Germany; ^4^ Max‐Planck‐Institut für Neurobiologie Martinsried Germany; ^5^ Nutritional Physiology Technical University of Munich Freising Germany; ^6^ Department of Medicine University of California, San Diego La Jolla CA USA

**Keywords:** calcium sensing receptor, dipeptide absorption, intestine, intracellular calcium signaling, peptide transporter 1

## Abstract

Although absorption of di‐ and tripeptides into intestinal epithelial cells occurs via the peptide transporter 1 (PEPT1, also called solute carrier family 15 member 1 (SLC15A1)), the detailed regulatory mechanisms are not fully understood. We examined: (a) whether dipeptide absorption in villous enterocytes is associated with a rise in cytosolic Ca^2+^ ([Ca^2+^]_cyt_), (b) whether the calcium sensing receptor (CaSR) is involved in dipeptide‐elicited [Ca^2+^]_cyt_ signaling, and (c) what potential consequences of [Ca^2+^]_cyt_ signaling may enhance enterocyte dipeptide absorption. Dipeptide Gly‐Sar and CaSR agonist spermine markedly raised [Ca^2+^]_cyt_ in villous enterocytes, which was abolished by NPS‐2143, a selective CaSR antagonist and U73122, an phospholipase C (PLC) inhibitor. Apical application of Gly‐Sar induced a jejunal short‐circuit current (*Isc*), which was reduced by NPS‐2143. CaSR expression was identified in the lamina propria and on the basal enterocyte membrane of mouse jejunal mucosa in both WT and *Slc15a1^−/−^* animals, but Gly‐Sar‐induced [Ca^2+^]_cyt_ signaling was significantly decreased in *Slc15a1^−/−^* villi. Clotrimazole and TRM‐34, two selective blockers of the intermediate conductance Ca^2+^‐activated K^+^ channel (IK_Ca_), but not iberiotoxin, a selective blocker of the large‐conductance K^+^ channel (BK_Ca_) and apamin, a selective blocker of the small‐conductance K^+^ channel (SK_Ca_), significantly inhibited Gly‐Sar‐induced *Isc* in native tissues. We reveal a novel CaSR‐PLC‐Ca^2+^‐IK_Ca_ pathway in the regulation of small intestinal dipeptide absorption, which may be exploited as a target for future drug development in human nutritional disorders.

## Graphical abstract

What is already known: The H^+^/dipeptide absorption rates in the small intestine depend on the maintenance of a negative membrane potential rather than the transmembrane proton gradient. What this study adds: The CaSR mediates intestinal dipeptide absorption through the Ca^2+^/IKCa/hyperpolarization mechanism Clinical significance: CaSR‐PLC‐Ca^2+^‐IKCa pathway could be exploited as a potential target in human nutritional disorders.

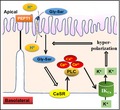

## 1 INTRODUCTION

After dietary proteins are digested to amino acids and di/tripeptides, they are absorbed into the intestinal epithelial cells (IEC) by a variety of amino acid transporters, and by the proton‐coupled di/tripeptide transporter peptide transporter 1 (PEPT1, also called solute carrier family 15 member 1 (SLC15A1) (Daniel, 2004; Shen & Matsui, 2018). Although PEPT1 is well characterized (Daniel & Zietek, 2016; Fei et al., 1994), its regulatory mechanisms remain incompletely understood (Spanier, 2014). Early experiments suggested that a proton gradient across the enterocyte luminal membrane may be an essential driving force for H^+^/dipeptide absorption (Ganapathy & Leibach, 1985; Shen & Matsui, 2018).

Chen et al. previously studied the dependence of murine jejunal dipeptide transport on luminal proton concentration, but they surprisingly found that dipeptides stimulate a similar H^+^/dipeptide absorption at low and high luminal pH, and that sustained H^+^/dipeptide absorption rates depend on the maintenance of a negative membrane potential rather than the transmembrane proton gradient. When the membrane depolarization by the inhibition of Na^+^/K^+^‐ATPase was initiated in mouse jejunum, a major reduction in Gly‐Sar‐induced *Isc* response was observed (Chen et al., 2010). In addition, apical Na^+^/H^+^ exchange (Kennedy, Leibach, Ganapathy, & Thwaites, 2002) and apical anion exchange (Simpson, Walker, Supuran, Soleimani, & Clarke, 2010) augment intestinal peptide absorption. Deductions from the regulation of other intestinal electrolyte and nutrient absorptive processes suggest that intracellular signaling‐dependent events may activate a variety of protein–protein interactions that may enhance the absorptive process, in particular Ca^2+^‐dependent processes such as IP_3_R‐binding protein released with inositol 1,4,5‐trisphosphate (IRBIT) translocation (He, Zhang, & Yun, 2008; He et al., 2015) or calcium‐sensing receptor (CaSR) activation (Macleod, 2013; Pacheco & Macleod, 2008; Tang et al., 2015b). It is unknown whether intestinal dipeptide absorption results in enterocyte Ca^2+^ signaling, whether PEPT1‐mediated dipeptide transport is involved in dipeptide‐elicited Ca^2+^ signaling and by which mechanisms this may occur, and what the consequences in the regulation of intestinal dipeptide absorption may be. Because Ca^2+^‐sensitive dyes were found to load poorly into native villous enterocytes of intact villi, Förster resonance energy transfer (FRET) was employed to assess changes in cytosolic free Ca^2+^ concentrations ([Ca^2+^]_cyt_) in native microdissected microvilli of the CAG‐TN‐XXL and Slc15a1^−/−^‐CAG‐TN‐XXL transgenic mouse, which encodes a genetically anchored calcium‐sensing protein TN‐XXL (Mank et al., 2008).

Under physiological conditions, various mechanisms contribute to the regulation of cellular and organ Ca^2+^ homeostasis. The CaSR is one of the most important regulators of Ca^2+^ homeostasis (Brown, 2013). Since the CaSR was first cloned from bovine parathyroid cells in 1993 (Brown et al., 1993), it has been reported to be widely expressed in multiple cell types of gastrointestinal (GI) tract (Chattopadhyay et al., 1998) and to involve in various roles of GI physiology (Chattopadhyay et al., 1998). CaSR can be activated by Ca^2+^, amino acids (L‐Ala, L‐Thr), peptides (Wang, Yao, Kuang, & Hampson, 2006), polyamines (spermine) (Quinn et al., 1997), and polycationic aminoglycoside antibiotics (Riccardi & Maldonado‐Perez, 2005). Activation of CaSR can stimulate phospholipase C (PLC)‐IP_3_ signaling pathway and prompt Ca^2+^ release from the endoplasmic reticulum (Hofer & Brown, 2003). The CaSR has been described to be expressed both in the apical and basolateral membrane of enterocytes and to be activated by a large variety of agonists including peptides (Chattopadhyay et al., 1998; Wang et al., 2006). In addition, the CaSR was recently identified as a modulator of intestinal nutrient and electrolyte absorption (Liu et al., 2018; Tang et al., 2015b).

Therefore, we investigated the involvement of the CaSR in dipeptide absorption and the underlying mechanisms. The dipeptide Gly‐Sar was chosen to the present study because it has been widely used to evaluate PEPT1‐mediated dipeptide transport (Alteheld et al., 2005; Buyse et al., 2001; Chen et al., 2010). Moreover, since [Ca^2+^]_cyt_ is a critical second cell messenger for the activation of Ca^2+^‐sensitive K^+^ channels, which are one of the key regulators for the maintenance of a negative membrane potential in IEC, we therefore wondered if K^+^ channels are involved in the luminal absorption of dipeptide; and if so, which type of K^+^ channels they are.

## 2 MATERIAL AND METHODS

### 2.1 Reagents and cell culture

Chemicals were obtained either from Sigma (Deisenhofen, Germany) or Merck (Darmstadt, Germany), if not indicated otherwise. Gly‐Sar, spermine, U73122 were purchased from Sigma (Deisenhofen, Germany). Apamin was purchased from Sigma (Shanghai, China). TRAM‐34 was purchased from MCE (Shanghai, China). NPS‐2143 was purchased from Tocris (Wiesbaden‐Nordenstadt, Germany). Iberiotoxin and Clotrimazole were purchased from Tocris (Shanghai, China). Mouse anti‐CaSR antibody was purchased from ThermoFisher (Waltham, USA). Polyclonal rabbit anti‐Actin antibody was purchased from Abcam (Cambridge, USA). The human colon cancer cell lines SW480 and SW620 were purchased from the Chinese Academy of Sciences in 2012. All cell lines were kept frozen in liquid nitrogen and after they were thawed, less than 20 of passages were used for 3 months in the present experiments.

### 2.2 Animal breeding

The CAG‐TN‐XXL transgenic mouse strain was generated by Prof. Oliver Griesbeck (Max‐Planck‐Institut für Neurobiologie, Germany) (Direnberger et al., 2012). The Pept1‐deficient mouse was generated by Hannelore Daniel (Department of Nutritional Physiology, Technical University of Munich, Germany) (Nässl et al., 2011), and was crossed with the CAG‐TN‐XXL transgenic mouse to obtain both *Slc15a1* ^−/−^ and *Slc15a1* ^+/+^ CAG‐TN‐XXL mice. The mice were bred in the animal research institute of Hannover Medical School on the C57/B6 background and were genotyped as recommended (Chen et al., 2010; Direnberger et al., 2012; Mank et al., 2008). Animal experiments followed the protocols approved by the Hannover Medical School and local authorities for the regulation of animal welfare (Regierungspräsidium). The mice were kept at a constant ambient temperature of 24 ± 1°C under a 12 hr light/dark cycle with free access to food and water before the experiments.

### 2.3 Assessing changes in enterocyte Ca^2+^ concentrations in isolated intestinal villi

In order to assess [Ca^2+^]_cyt_ in jejunal villous enterocytes after Gly‐Sar exposure, the proximal jejunum ( approximately 6 cm distal to the pylorus) was removed and immediate placed in ice‐cold Ringer's solution (solution composition in mM: 147 NaCl, 4 KCl, 2.2 CaCl_2_ with 500 µM DTT to prevent the mucus to clog the villi, pH 7.4). The jejunum was sliced into 0.5 cm sections and each section was opened along the mesenteric border. One piece of tissue was transferred on the cooled stage of a dissecting microscope, and individual villi were detached from the intestine by snapping them off from the mucosa with sharpened microdissection tweezers. Care was taken not to damage the apical part of the villi. The villi were attached to a glass coverslip coated with Cell‐Tak adhesive. The villi were fixed on the coverslip with a polycarbonate membrane (25 mm diameter, pore size 3 µm, Osmonics) in a custom‐made perfusion chamber, and perfused with prewarmed (37°C) O_2_‐gassed buffer A (solution composition in mM: 130 Nacl, 10 HEPES, 5 Tris, 2.25 KH_2_PO_4_, 1.2 MgSO_4_, 1.2 Ca‐Gluconate, pH7.4). Buffer B, in which 20 mM Gly‐Sar, replaced 10 mM NaCl. The FRET‐based biosensor TN‐XXL composed of two fluorescent proteins, the cyan fluorescent protein (CFP) and the Citrine cp174 acting as donor (D) and acceptor (A), respectively. Ca^2+^‐imaging was done at a Zeiss LSM 780 with the acquisition settings as follows: 40x/1.2 NA water immersion objective, excitation 440 nm, online fingerprinting mode, reference spectra obtained from single fluorophores, 300 time steps, 10s interval. The acceptor/donor ratio (A/D ratio) was used as a readout of the TN‐XXL as measure for [Ca^2+^]_cyt_.

### 2.4 Ussing chamber experiments

Ussing chamber experiments were performed as previously described (Chen et al., 2010). The jejunal mucosa was mounted between chambers with an exposed area of 0.625 cm^2^. Parafilm “O” rings were used to minimize edge damage to the tissue where it was secured between the chamber halves. Transepithelial short‐circuit current (*Isc*; reported as µEq cm^–2^ h^–1^) was measured via an automatic voltage clamp (voltage–current clamp EVC‐4000; World Precision Instruments) After a 30 min measurement of basal parameters, the Gly‐Sar (20 mM) or control vehicle was added to the mucosa side of tissue in Ussing chambers. When the inhibitor was used, it was added to the serosal side, mucosa side and both sides of tissue at 30 min before the Gly‐Sar. Tissues were bathed with solutions containing HCO_3_^−^ (solution composition in mM: 108 NaCl, 22 NaHCO_3_, 3 KCl, 1.3 MgSO_4_, 2 CaCl_2_, 1.5 KH_2_PO_4_, 2 CaCl_2_, 1.5 KH_2_PO_4_ and pH 7.4) in a 95% O_2_ /5% CO_2_ atmosphere.

### 2.5 Immunofluorescence staining

Immunofluorescence staining was carried out as described previously (Chen et al., 2010). Briefly, the slides with mouse jejunum from C‐57 mouse were incubated with an anti‐primary antibodies incubated overnight at 4°C. Thereafter, followed by a second antibody and was incubated for 1h at room temperature. The tissues was incubated with Alexa Fluor 633 phalloidin. The nuclei are stained using slow fade with DAPI. Samples were imaged on the confocal microscope (a Leica DM IRB with a TCS SP2 AOBS scan).

### 2.6 Quantitative real‐time PCR

Real‐time PCR reactions were carried out using Rotor‐Gene SYBR Green PCR Kit in the Rotor‐Gen Q Cycler (Qiagen) were performed as described previously (Luo et al., 2017; Yeruva et al., 2015). PCR extensions were performed at 60°C with 40 repeats. Data were analyzed using Rotor‐Gene Software and exported to Microsoft Excel. Values within the log‐linear phase of the amplification curve were defined for each probe/primers set and analyzed using the ^ΔΔ^Ct method. The primers for mRNA expression analysis were listed in Table 1.

**Table 1:** List of primer sequences

Genotpying	Sequence	Product length, bp
BKCa.for	5′‐TTC CTC AGC AAT CAG AGC CTC‐3′	91
BKCa.rev	5′‐ACA GCA TTT GCC GTC AGT GTC‐3′	
IKCa.for	5′‐GTT CTA CAA ACA TAC TCG CAG GA‐3′	82
IKCa.rev	5′‐GCG TGT CAA TCT GTT TCT CAA‐3′	
SKCa.for	5′‐GAT TGA CCA TGC CAA AGT GAG‐3′	103
SKCa.rev	5′‐ACA TGA CAT TCT GCA TCT TGG‐3′	
Actin(mouse).for	5′‐AGA GGG AAA TCG TGC GTG AC‐3′	138
Actin(mouse).rev	5′‐CAA TGA TGA TGA CCT GGC CGT‐3′	

Note

The details of primer sequence, product length and the accession number of different genes that we used.

### 2.7 Western blotting

Tissues and cells were lysed with lysis buffer and centrifuged at 12,000× *g* for 15 min to remove insoluble material. For immunoprecipitation studies, lysates were incubated with a CaSR antibody for 1 hr at 4°C, followed by another 1 hr incubation with Protein A‐agarose beads at 4°C. Proteins were eluted with 2× loading buffer, boiled for 5 min, and separated by SDS‐PAGE (10%). Resolved proteins were transferred onto a PVDF membrane (Millipore Corporate). Membranes were blocked by 5% blocking buffer, followed by incubation with primary antibodies as indicated overnight. After washing with TBST, HRP conjugated secondary antibody was applied for 1hour at room temperature. The signals were visualized using enhanced chemiluminescence (GE healthecare Life Sciences).

### 2.8 Statistical analysis

All data are calculated as the means for a series of n experiments ± *SEM*. Data were analyzed by one‐way ANOVA followed by the Student–Newman–Keul post hoc test or by Student's *t* tests for paired or unpaired samples with GraphPad Prism 5.0. *p* < .05 was considered statistically significant.

## 3 RESULTS

### 3.1 Evaluation of [Ca^2+^]_cyt_ response to Gly‐Sar in villous enterocytes

**Figure 1:**
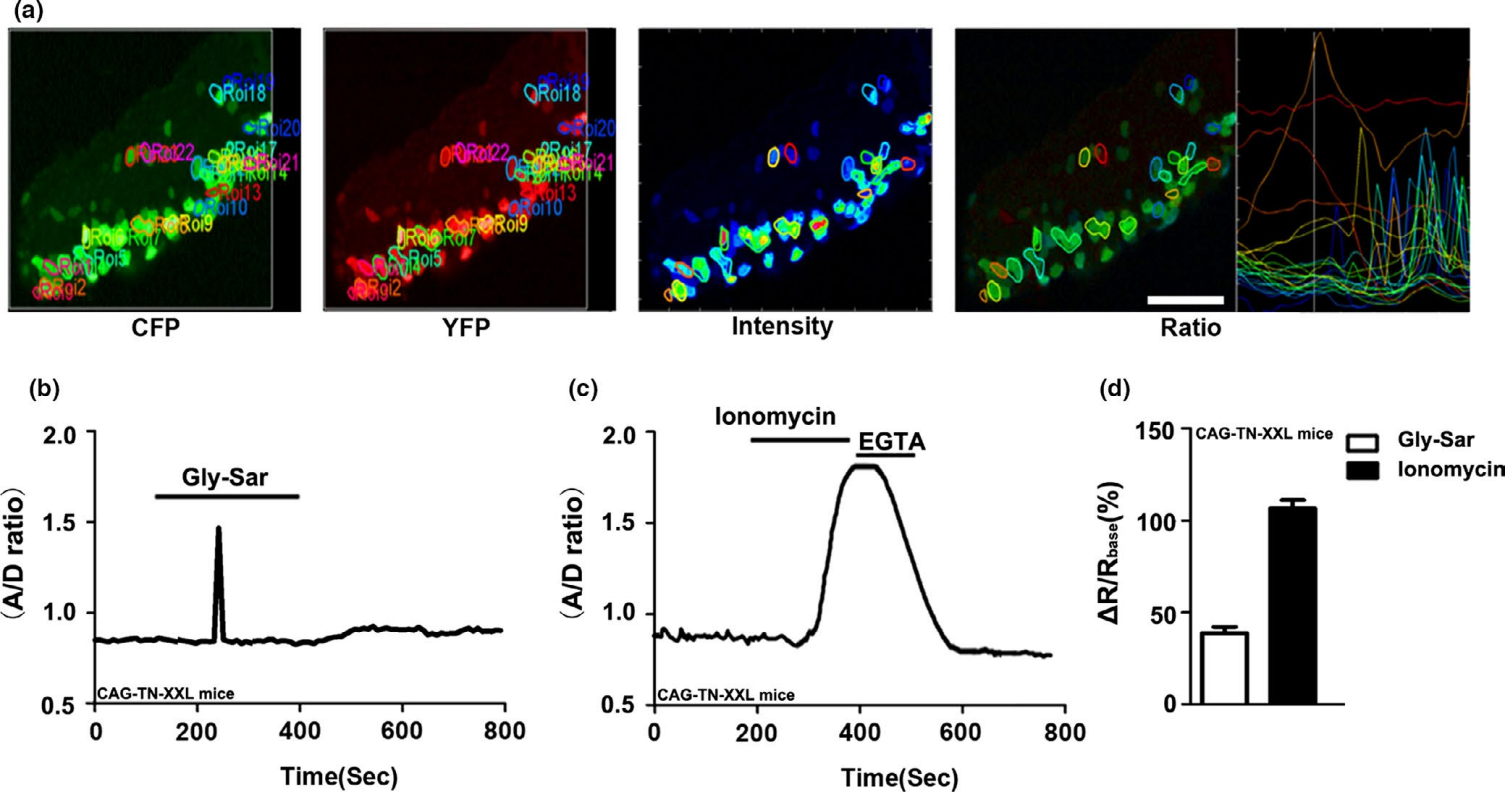
Calcium imaging in villous enterocytes isolated from the jejunum of CAG‐TN‐XXL mice. (a) Ca^2+^ imaging taken from the enterocyte regions of interest (ROIs) and the transient calcium changes were restricted to an individual enterocyte (scale bar = 60 μm). (b) Representative trace of variations in [Ca^2+^]_cyt_ was detected following perfusion with Gly‐Sar (20 mM) in villous enterocytes. (c) Marked changes in [Ca^2+^]_cyt_ signaling were measured after treatment with ionomycin (10 μM) in villous enterocytes. (d) The summary on the ratio change (ΔR: the maximal ratio after addition of Gly‐Sar or ionomycin minus the ratio before addition), *n* = 7

**Figure 2:**
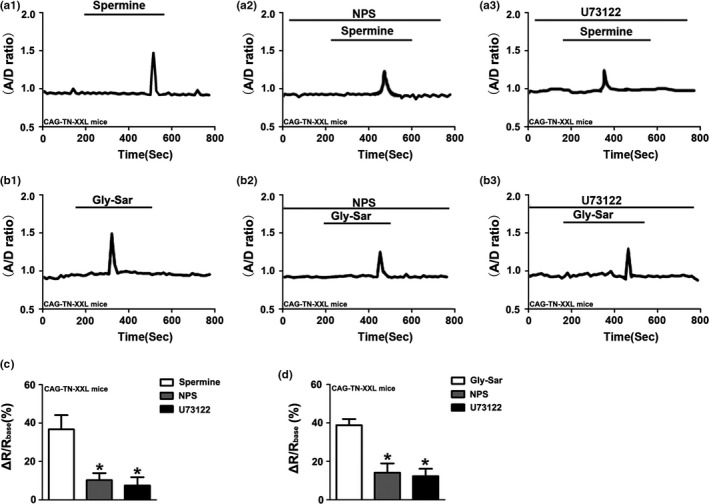
CaSR stimulation results in an increase in [Ca^2+^]_cyt_ in villous enterocytes dissected from the jejunum of CAG‐TN‐XXL mice. (a) The effect of spermine (SP, 3 mM) on [Ca^2+^]_cyt_ in villous enterocytes in the absence (a_1_) or the presence of NPS‐2143 (30 μM, a_2_), or U73122 (20 μM, a_3_). (b) The effect of Gly‐Sar (20 mM) on [Ca^2+^]_cyt_ in villous enterocytes in the absence (b_1_) or the presence of NPS‐ 2,143 (30 μM, b_2_) and U73122 (20 μM, b_3_). (c and d) The summary on the ratio change of spermine‐ and Gly‐Sar‐stimulated calcium response in the absence or the presence of NPS‐2143 and U73122. Data are the mean ± *SEM* from three independent experiments. *p < .05 versus. the controls

Since native enterocytes within microdissected villi displayed strong autofluorescence when excited in the blue spectrum, and loaded poorly with the Ca^2+^‐sensitive dye Fura‐2, the CAG‐TN‐XXL mouse, which expresses a genetically encoded FRET‐based biosensor TN‐XXL was used to study Gly‐Sar elicited alterations in [Ca^2+^]_cyt_ (Direnberger et al., 2012; Mank et al., 2008). We previously suggested that 20 mM was the best concentration of Gly‐sar in the experiemnts, as this mimics the conditions of proteins after their digestion and hydrolysis in the small intestine (Chen et al., 2010). Therefore, 20 mM Gly‐sar was used in all following experiments. Figure 1 showed that Gly‐Sar (20 mM) stimulated an increase in [Ca^2+^]_cyt_ (represented as A/D ratio) in villous enterocytes isolated from the jejunum of CAG‐TN‐XXL mouse (Chen et al., 2010). Consistently, ionomycin, a well‐known Ca^2+^ ionophore, also caused a marked and long‐lasting increase in [Ca^2+^]_cyt_ which was reverted by adding the Ca^2+^‐chelator EGTA. These data suggest that Gly‐Sar addition may induce an increase in [Ca^2+^]_cyt_ in villous enterocytes. Spermine (3mM), a commonly used CaSR activator, also caused a strong increase in [Ca^2+^]_cyt_, which was significantly inhibited by the CaSR inhibitor NPS‐2143 (30 μM), and U73122 (20 μM), a widely employed PLC inhibitor(Hou et al., 2004; Macmillan & McCarron, 2010) (Figure 2a,c). Gly‐Sar induced increase in [Ca^2+^]_cyt_ was also significantly blocked by NPS‐ 2143 and U73122 (Figure 2b,d). These experiments suggest that dipeptide absorption is accompanied by CaSR activation‐induced Ca^2+^ signaling in villous enterocytes.

### 3.2 Role of PEPT1 in maintaining the [Ca^2+^]_cyt_ response in villous enterocytes

**Figure 3:**
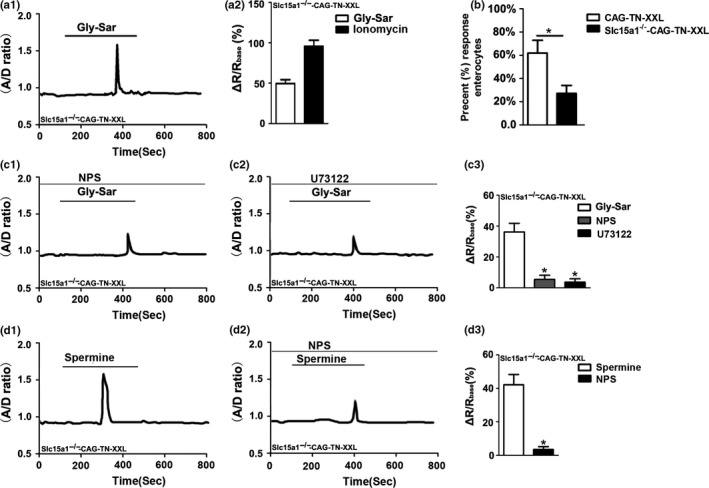
Gly‐Sar‐induced [Ca^2+^]_cyt_ increase is diminished in villous enterocytes dissected from the jejunum of *Slc15a1^−/−^*‐CAG‐TN‐XXL mice. a_1_: Traces of calcium responses was detected following perfusion with Gly‐Sar (20 mM) in villous enterocytes. a_2_: The summary on the ratio change (the ratio after addition of Gly‐Sar or ionomycin minus the ratio before addition), *n* = 7. (b) Summarized data show the percentage of responsive enterocytes in each villi after addition of Gly‐Sar. The values are expressed as the mean ± *SEM* in groups of 5 mice (1 to 2 isolated villi were cut from each mouse and in each villi there were approximately 10 enterocytes, which were chosen at random). *p < .05 versus. CAG‐TN‐XXL. (c) The effect of Gly‐Sar (20 mM) on [Ca^2+^]_cyt_ in villous enterocytes in the absence or the presence of NPS‐ 2,143 (30 μM, c_1_) and U73122 (20 μM, c_2_). (c_3_) The summary on the delta ratio of Gly‐Sar‐stimulated calcium response in the absence or the presence of NPS‐2143 and U73122. (d) The effect of spermine (3 mM) on [Ca^2+^]_cyt_ in villous enterocytes in the absence (d_1_) or the presence of NPS‐2143 (30 μM, d_2_). (d_3_) The summary on the ratio change of spermine‐stimulated calcium response in the absence or the presence of NPS‐2143. Data are the mean ± *SEM* from three independent experiments. *p < .05 versus the controls

PEPT1 is the major apical transporter for di‐ and tripeptides in the intestinal brush border membrane, but a certain fraction of a luminal peptide load can enter the systemic circulation in a PEPT1‐independent manner (Hu et al., 2008). In order to assess whether the expression of PEPT1 is important for the generation of the Gly‐Sar induced Ca^2+^ response, the *Slc15a1^−/−^*‐CAG‐TN‐XXL mouse was used. The [Ca^2+^]_cyt_ measurements were performed in vitro as described for the experiments in Figure 3a, by superfusing the villi with solutions with 20 mM Gly‐Sar. We found that in the *Slc15a1^−/−^*‐CAG‐TN‐XXL mouse, both Gly‐Sar and ionomycin caused a rapid increase in the A/D ratio, but the percentage of reactive villous enterocytes was significantly decreased in the *Slc15a1^−/−^*‐CAG‐TN‐XXL mouse compared to that in control mice (CAG‐TN‐XXL) (Figure 3b). However, the amplitudes of the signals were comparable in *Slc15a1* ^+/+^‐CAG‐TN‐XXL and *Slc15a1^−/−^*‐CAG‐TN‐XXL mice (Figure S1). As in the *Slc15a1* ^+/+^ villi, the Gly‐Sar induced increase in [Ca^2+^]_cyt_ was blocked by NPS‐2143 (30 μM), and U73122 (20 μM) (Figure 3c). Spermine (3 mM) caused a similar calcium increase in the *Slc15a1^−/−^*‐ as in the *Slc15a1* ^+/+^‐CAG‐TN‐XXL mouse, which was significantly inhibited by NPS 2143 (30 μM) (Figure 3d). These data demonstrate that the presence of PEPT1 is important for the CaSR‐induced [Ca^2+^]_cyt_ increase, but that Gly‐Sar also has an effect on [Ca^2+^]_cyt_ in the absence of PEPT1 expression. It is likely that at the rather high concentrations used to saturate the PEPT1 transporter, a significant fraction of the Gly‐Sar permeates via the tight junctions, but transcellular transcytosis is also a highly active transport pathway in the intestine (Lundquist & Artursson, 2016). Both PEPT‐1 dependent and –independent increase in [Ca^2+^]_cyt_ was inhibited by CaSR inhibition.

### 3.3 Involvement of CaSR in Isc response to the dipeptide in mouse jejunum

**Figure 4:**
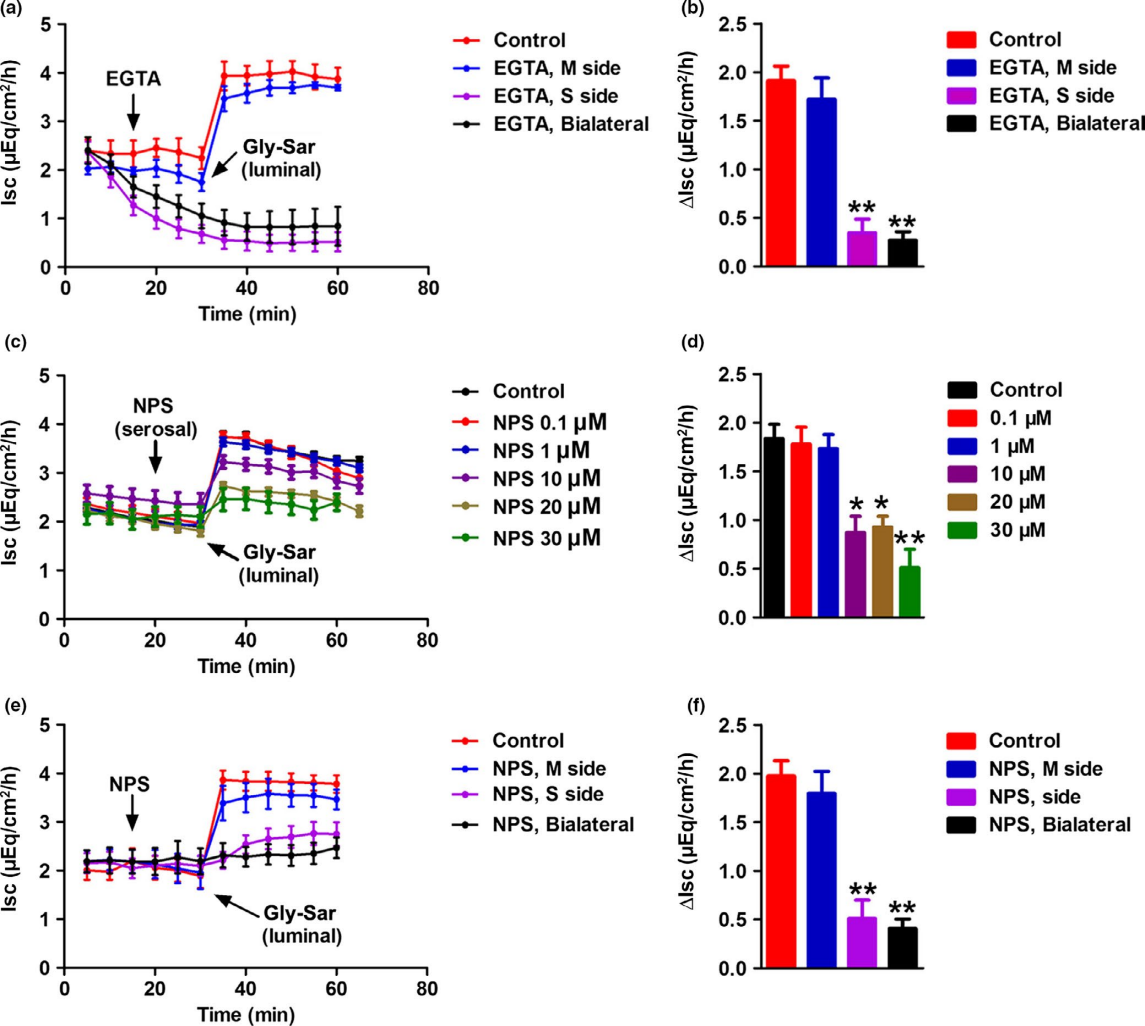
Effects of EGTA and NPS‐2143 on Gly‐Sar‐stimulated jejunal *Isc* response in mice. (a) Time course of Gly‐Sar‐induced jejunal *I*sc when extracellular Ca^2+^ was omitted from single side or both sides of the jejunal tissues. In the control, CaCl_2_ (2 mM) existed on both sides of the tissues, but Gly‐Sar (20 mM) was added to mucosal side. In the experimental series, Gly‐Sar was also added to mucosal side after extracellular Ca^2+^ omission plus EGTA (0.1 mM) from different sides for 20 min. (b) Summary data comparing the effects of Ca^2+^ omission plus EGTA on Gly‐Sar induced increase in Isc (Δ*Isc*). (c) Time courses of Gly‐Sar induced jejunal *I*sc at doses of 0.1‐30μM. (d) Gly‐Sar induced a dose‐dependent increase in net peak jejunal *I*sc. (e) Time course of Gly‐Sar induced jejunal *I*sc in the absence or the presence of NPS‐2143 on single side or both sides of the jejunal tissues. Gly‐Sar was added to mucosal side after NPS‐2143 (30 μM) was added to different sides of the tissues for 20 min. (f) Summary data comparing the effects of NPS‐2143 on Gly‐Sar induced ∆*I*sc peak. Data are the given as mean ± *SEM* (*n* = 6 in each series), *p < .05, **p < .01 versus control

Since [Ca^2+^]_cyt_ plays a critical role in modulating intestinal epithelial ion transports (Cheng, 2012), we conducted Ussing chamber experiments to test whether [Ca^2+^]_cyt_ is involved in jejunal dipeptide absorption. Early studies in intact isolated intestinal mucosa in Ussing chamber systems demonstrated the dipeptide transport is always accompanied by the *Isc* (Addison, Burston, & Matthews, 1972; Clarke, 2009; Larsen, Mertz‐Nielsen, Hansen, Poulsen, & Bindslev, 2001). As shown in Figure 4a, Gly‐Sar (20 mM) induced a time‐dependent increase in jejunal *Isc* in the presence of external Ca^2+^ (2 mM). However, Ca^2+^ removal (Ca^2+^ omission plus 100 μM EGTA) from serosal side or both sides abolished Gly‐Sar‐stimulated jejunal *Isc* (Δ*Isc*, the difference between the baseline and the peak value at 5 min after addition of the drug). In contrast, Ca^2+^ removal from mucosal side failed to alter Gly‐Sar‐induced *Isc* (Figure 4a&B)*.* These data demonstrate an important role of external Ca^2+^ on the serosal side in the regulation of enterocyte dipeptide absorption. Since external Ca^2+^ is the CaSR activator, we further examined if the CaSR is involved in this process. Different concentrations (0.1–30 μM) of NPS‐2143, a selective CaSR antagonist (Gwynne, Ly, Parry, & Bornstein, 2017; Zhou & Pestka, 2015), were added to the serosal side of the tissue. As shown in the time course of jejunal *Isc* (Figure 4c,d), Gly‐sar induced a significant increase in *I*sc, which was progressively smaller when the tissue was preincubated with NPS‐2143 in the serosal solution in a dose‐dependent manner. In contrast, addition of NPS‐2143 in mucosal side did not evoke any obvious effect (Figure 4e,f). Therefore, activation of CaSR on serosal side is likely involved in the *Isc* response to the dipeptide in mouse jejunum.

### 3.4 Expression of the CaSR in mouse jejunal mucosa

**Figure 5:**
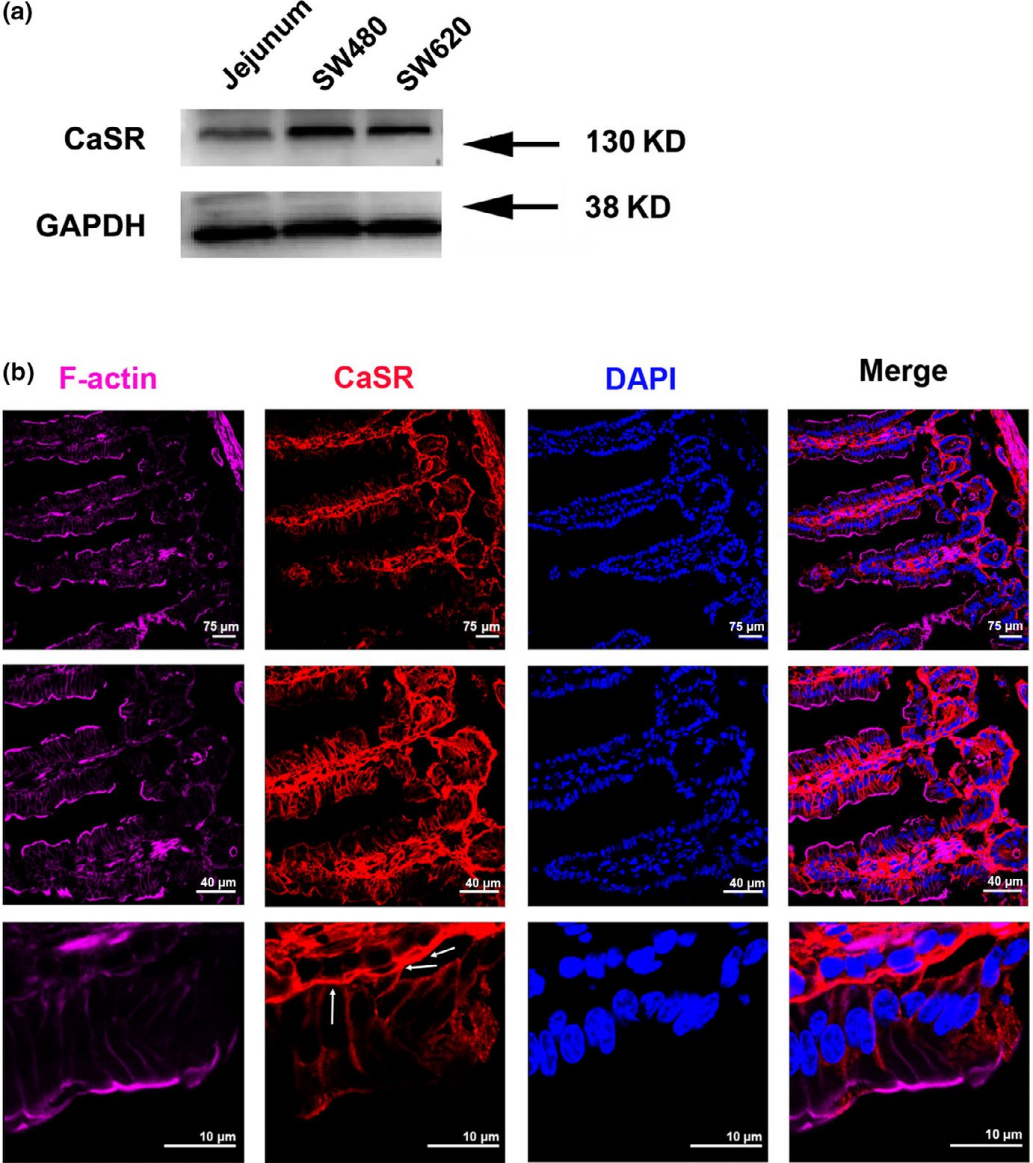
The expression of CaSR in mouse jejunum mucosal tissues. (a) After mouse jejunum mucosal tissues, SW‐480 cells, and SW‐620 cells were lysed, Western blot analysis was performed to detect protein expression of the CaSR using a specific anti‐CaSR monoclonal antibody. GAPDH was used as a loading control (*N* = 3). (b) The immunofluorescence staining of mouse jejunum mucosa showing the immunoreactivity of F‐actin (purple), CaSR (red) and DAPI (blue) in the villi and crypts of the jejunum mucosa. Note that intense CaSR staining is evident in the enterocytes, basolateral and plexi. These data are representative of three experiments with similar results

A specific antibody was used to examine the expression and distribution of CaSR in the jejunal mucosa by Western blot and immunofluorescence staining, respectively (Xie et al., 2014). CaSR expressions were detected in the mouse jejunal mucosa and human colon cancer cells SW480 and SW620 (Quinn et al., 1997) (Figure 5a). The distribution of CaSR was also identified in intestinal epithelial cells through immunofluorescence staining. Figure 5b shows images of the typical villous cells in mouse jejunal mucosa with different magnifications. The CaSR was found to primarily locate on the basolateral side of the villous cells and submucosal neurovascular structures. The localization of CaSR on the basolateral membrane of enterocytes, consistently with a previous report (Chattopadhyay et al., 1998). And further supports our notion described earlier that CaSR activation on serosal side is involved in jejunal *I_sc_* response to the dipeptide.

### 3.5 Effects of K_Ca_ channel blockers on Gly‐Sar‐induced Isc

**Figure 6:**
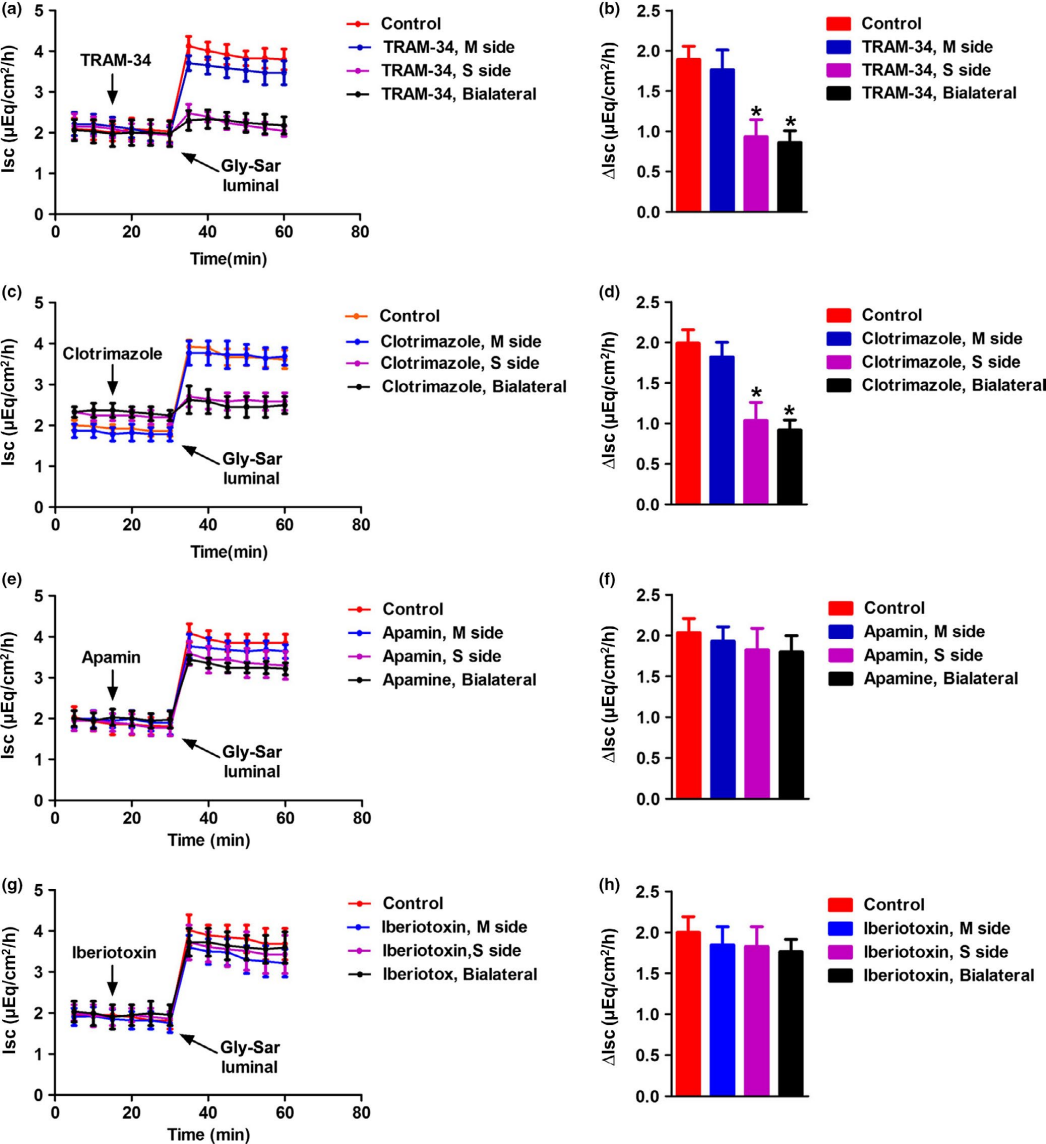
Effects of K_Ca_ channel blockers on Gly‐Sar‐stimulated jejunal *Isc* response in mice. Inhibitory effect of TRAM‐34, Clotrimazole, lberiotoxin and Apamin on the time course of Gly‐Sar stimulated jejunal mucosal *I*sc (a–d). Gly‐Sar was added to mucosal side after TRAM‐34 (10 μM) Clotrimazole (30 μM), lberiotoxin (100 nM) or Apamin (100 nM) were added to different sides of the tissues for 20 min (e–h). (d, f and h) Summarize the data comparing the effects of Ca^2+^ omission plus TRAM‐34, Clotrimazole, lberiotoxin and Apamin on Gly‐Sar‐induced ∆*I*sc. Data are given as mean ± *SEM* (*n* = 5 in each series), *p < .05 versus control

The jejunal mucosal tissues were equilibrated in Ussing chambers for 30 min, basal *Isc* was recorded for an additional 30 min. Subsequently, jejunal mucosal tissues were pretreated with different K_Ca_ channel blockers, or their vehicle (DMSO or distilled water), to both sides of the tissues. As shown in Figure 6. TRAM‐34 (10 μM) and Clotrimazole (30 μM), both of them selective blocker of Ca^2+^‐activated K^+^ channel (IK_Ca_), significantly inhibited the Gly‐Sar‐induced *Isc*. However, neither Iberiotoxin (100 nM) a selective blocker of large‐conductance K^+^ channel (BK_Ca_), nor Apa min (100 nM) a selective blocker of small‐conductance K^+^ channel (SK_Ca_) evoked any obvious effect. These data suggest that IK_Ca_ rather than BK_Ca_ and SK_Ca_ are involved in H^+^/dipeptide absorption.

### 3.6 mRNA expression of K_Ca_ channels in jejunal epithelium

**Figure 7:**
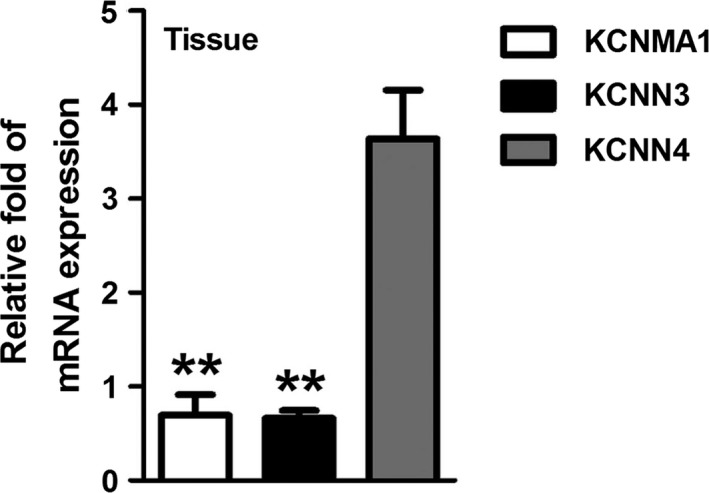
Comparison on the mRNA expression of different subtypes of K_Ca_ channels in mouse jejunum mucosa. The figure represents the relative fold changes in the mRNA expression of different subtypes of K_Ca_ channels, KCNMA1, KCNN3, and KCNN4 in mouse jejunum. (**p < .01, vs. KCNN4, *n* = 3, these data are representative of 3 experiments conducted on different mice)

Three different subtypes of K_Ca_ channels, BK_Ca_ (KCNMA1), SK_Ca_ (KCNN3), and IK_Ca_ (KCNN4), have been identified to be expressed in gastrointestinal epithelia and involved in intestinal absorptive and ion transport (Bleich et al., 1996; Bowley, Morton, Hunter, & Sandle, 2003; Joiner et al., 2003; Warth, 2003). To investigate whether these K_Ca_ subtypes are also expressed in the jejunum, the expression of mRNA specific for the three types of these channels was measured by qPCR. Figure 7 shows that the mRNA expression of KCNMA1, KCNN3 and KCNN4 in mouse jejunal mucosa. The data showed that KCNN4 amplification products were much higher than the KCNMA1 and KCNN3 in tissues, corresponding to previous reports (Dong, Smith, Hovaida, & Chow, 2006). Therefore, on the basis of the mRNA expression *pattern*, KCNN4 is the most likely candidate involved in the regulation of H^+^/dipeptide absorption, which is consistent with our functional data that IK_Ca_ plays a major role in this process.

## 4 DISCUSSION AND CONCLUSION

The proton‐coupled di/tripeptide transporter PEPT1 has been accepted as the only intestinal peptide transporter, and is responsible for a significant part of protein‐associated calorie uptake. PEPT1 can also transport a vast amount of substrates, including drugs and bacterial products, and may thus be both of important pathophysiological consequence, as well as an interesting target for drug design. Although much has been learned regarding its transcriptional regulation of PEPT1 expression and the influence of hormones and intracellular kinases (Spanier & Rohm, 2018), less is known about the trafficking, membrane retention, the interaction with other transporters, and the ionic requirements for sustained H^+^/dipeptide absorption (Spanier & Rohm, 2018).

Early studies suggested that a proton gradient, possibly maintained by the activity of luminal Na^+^/H^+^ exchangers, is an essential driving force for H^+^/dipeptide uptake (Ganapathy & Leibach, 1985). However, while the abolition of a transapical protein gradient did not have a major effect on the rate of H^+^/dipeptide absorption, apical membrane depolarization strongly reduced H^+^/dipeptide absorption (Chen et al., 2010). Since the process of H^+^/dipeptide uptake by PEPT1 depolarizes the membrane (Matsumura, Miki, Jhomori, Gonoi, & Seino, 2005; Vig et al., 2006), it is not surprising that mechanisms are required to maintain the negativity of the membrane during H^+^/dipeptide absorption. Similar findings have been obtained in oocytes expressing PEPT1 in which low pH was shown to affect transport only at low but not at high substrate concentrations. Those data suggested that the inside negative membrane potential could increase the Vmax PEPT1 (Kottra & Daniel, 2001; Mackenzie et al., 1996). The basolateral Na^+^/K^+^ ATPase is one such mechanism (Chen et al., 2010), but the molecular nature of the involved K^+^ channels has not been investigated.

In addition to the general mechanisms required for any persistent cation‐coupled nutrient uptake, namely the activity of the Na^+^/K^+^ ATPase and of basolateral K^+^ channels through which a negative membrane potential is maintained despite the persistent depolarizing effect of the ongoing electrogenic cation import, a large variety of receptor‐, second messenger‐ and protein–protein interaction events have been delineated that stimulate trafficking to the apical membrane, augment the interaction of electrolyte and nutrient transporters, and enhance intestinal nutrient and fluid absorption. In fact, a stimulatory network of signaling of glucose and amino acids or peptides through taste receptors that regulate the apical availability/function of glucose and peptide/amino acid transporters via Ca^2+^‐signaling has been postulated, but data on the actual [Ca^2+^]_cyt_ during dipeptide absorption in enterocytes do not exist (Daniel & Zietek, 2016; Mace et al., 2010).

Our first question was therefore whether dipeptide absorption elicits a rise in [Ca^2+^]_cyt_ in native jejunal enterocytes. Because our attempts to reliably measure changes in intracellular Ca^2+^ levels in native enterocytes with fluorescent dye loading was hampered by poor loading through the enterocyte apical membrane, potential interferences of this low signal with the change in intracellular pH value (pHi) that occur during H^+^/dipeptide uptake, and the strong autofluorescence of enterocytes in the blue spectral range. We therefore made use of the CAG‐TN‐XXL mouse, which expresses a genetically encoded FRET‐based biosensor for Ca^2+^, namely a genetically optimized troponin C, TN‐XXL to display an increased signal strength in the low‐calcium regime (Geiger et al., 2012; Mank et al., 2008). The CAG‐TN‐XXL mouse displays expression of the TN‐XXL in the intestine (Direnberger et al., 2012), but we noticed that the expression of TN‐XXL in the intestinal enterocytes declines strongly with age, and the experiments need to be done in villi of adolescent mice. The acceptor/donor ratio provides a semiquantitative assessment of [Ca^2+^]_cyt_ and demonstrated a Gly‐Sar induced increase in [Ca^2+^]_cyt_. Experiments designed to understand the origin of this Ca^2+^ signaling showed that the CaSR activation with spermine also elicited a rise in [Ca^2+^]_cyt,_ and both the Gly‐Sar and the spermine‐induced Ca^2+^‐signaling were attenuaed by the CaSR inhibitor.

The CaSR, one member of the G protein‐coupled receptor (GPCR) family, plays a critical role in maintaining Ca^2+^ homeostasis. The CaSR is widely distributed in various human organs, including the entire gastrointestinal tract (Geibel & Hebert, 2004). It can be activated by Ca^2+^, amino acids (L‐Ala, L‐Thr), peptides (Wang et al., 2006), polyamines (spermine) (Quinn et al., 1997). Activation of the CaSR has been shown to increase [Ca^2+^]_cyt_ in many types of mammalian cells (Hofer & Brown, 2003). CaSR stimulation has been shown to reverse the colonic fluid secretory effect of enterotoxins by enhancing the phosphodiesterase‐mediated destruction of cyclic nucleatides (Geibel et al., 2006). CaSR stimulation also results in an increase in apical Cl^−^/HCO_3_^−^ and SCFA/HCO_3_^−^ exchange in the colon, which enhances fluid absorption (Tang et al., 2015a). In addition, CaSR stimulation elicits gastrointestinal hormone release and has an anti‐inflammatory action in the colon (Tang et al., 2016). It also is involved in hVD3‐induced duodenal Ca^2+^ absorption (Fleet & Schoch, 2010).

In this study, we also observed that the jejunal *I*sc response to Gly‐Sar was significantly reduced by the exclusively serosal addition of either Ca^2+^ chelator EGTA or CaSR antagonist. This suggests that the stimulation of the CaSR by addition of Gly‐Sar to the luminal bath occured beyond the enterocyte brush border membrane, in which PEPT1 resides. Intestinal CaSR expression has been localized to the apical and basolateral membrane of enterocytes (Cheng, Okuda, Hall, Geibel, & Hebert, 2002). However, we could only detect CaSR immunostaining in the basal membrane and in cellular elements of the lamina propria (Figure 5). This is consistent with the exclusive effect of the CaSR.

Gly‐Sar is a nonhydrolyzable dipeptide and therefore exclusively absorbed into enterocytes by PEPT1. Of the di‐ and tripeptides absorbed during a meal by PEPT1, the majority are further hydrolyzed and exported as aminoacids, but a fraction is also exported as dipeptides by as yet molecularly undefined carriers (Terada & Inui, 2004), In order to get more insight into the mechanisms we carried out studies in *Slc15a1^−/−^*‐CAG‐TN‐XXL mouse, the percentage of reactive villous enterocytes were significantly decreased compare to CAG‐TN‐XXL. Previous reports suggested that 70% uptake of Gly‐Sar was via transecellular pathway in WT mice, the remaining 30% could be absorbed via paracellular pathways, passive diffusion or endocytosis (Chen et al., 2010; Hu et al., 2008). However, in the *Slc15a1^−/−^*‐CAG‐TN‐XXL mouse, we still observed calcium response, suggesting that CaSR is also involved in PEPT1‐independent increase of cytosolic calcium by Gly‐Sar. Wenzel et al. reported that PEPT1‐mediated cefixime uptake into human intestinal epithelial cells was increased by nifedipine, a putative CaSR agonist (Wenzel, Kuntz, Diestel, & Daniel, 2002), suggesting a modulation of PEPT1 by CaSR (although they interpreted their findings differently at the time).

**Figure 8:**
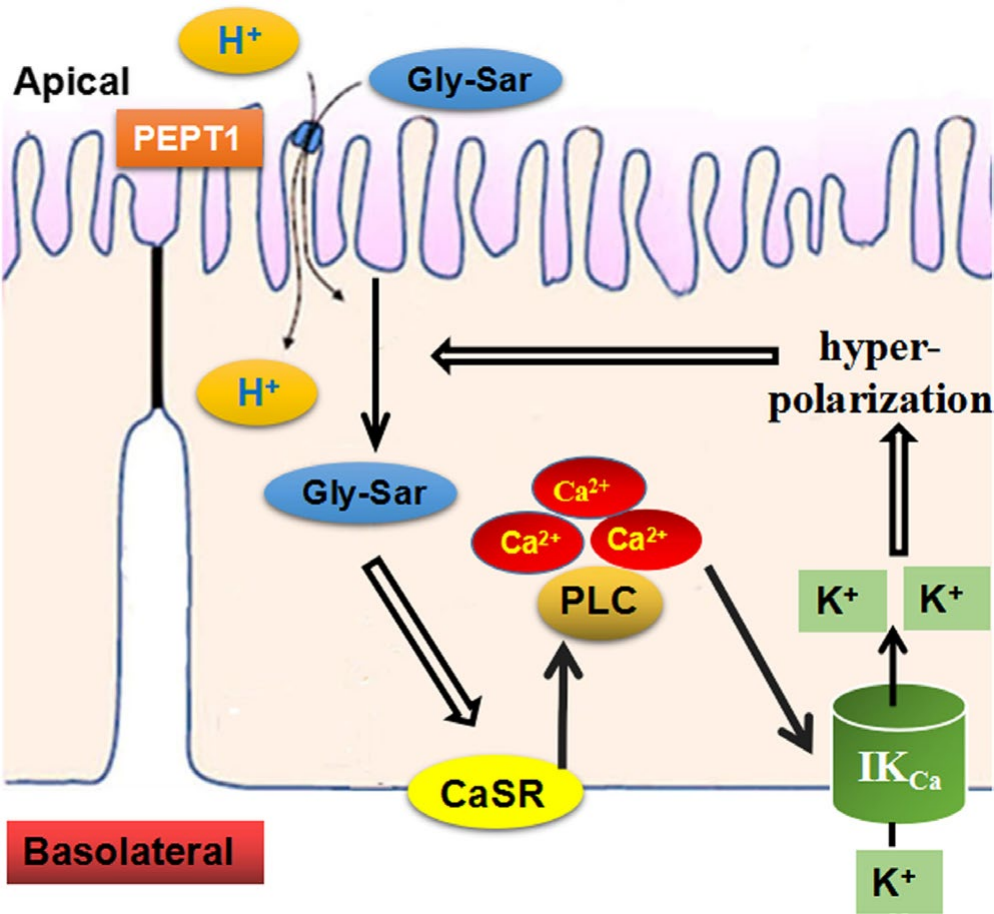
Schematic for CaSR‐PLC‐Ca^2+^‐IK_Ca_‐mediated dipeptide absorption into small intestinal epithelial cells. Luminal Gly‐Sar and protons are co‐transported into enterocytes via the PEPT1. The absorbed Gly‐Sar stimulates the CaSR located on the basolateral side of enterocytes, leading to the activation of phospholipase C (PLC) and increase in [Ca^2+^]_cyt_ that activates the IK_Ca_. IK_Ca_ opening induces cell hyperpolarization, providing a driving force for transepithelial Gly‐Sar absorption through PEPT1. This constitutes a positive feedback loop to promote further intestinal absorption of dipeptides

It is unclear which mechanism is responsible for the importance of CaSR‐mediated signaling for Gly‐Sar uptake. Earlier work has demonstrated that the H^+^‐coupled electrogenic dipeptide uptake in the native small intestine mediated by PEPT1 was solely dependent on membrane potential, irrespective of extracellular pH. It is well known that the opening of the K^+^ channels lead to hyperpolarization of the cells, providing a driving force for HCO_3_^‐^ secretion, dipeptide absorption and Cl^‐^/HCO_3_^‐^ exchangers, moreover keeping the potassium channel open requires only a brief calcium transient signal (Chen et al., 2010; Hogan et al., 1997; Seidler et al., 2010; Spiegel et al., 2003). Our Ussing chamber study showed that blockade of IK_Ca_ channels significantly inhibited Gly‐Sar‐induced *Isc,* indicating that the IK_Ca_ channels are involved in the Gly‐Sar absorption. IK_Ca_ channels are functionally expressed on the basolateral side of duodenal epithelial cells and regulate intestinal transepithelial Cl^‐^ and HCO_3_^‐^ secretion (Dong et al., 2006). We speculate that an increase in [Ca^2+^]_cyt_ may induce hyperpolarization via stimulation of IK_Ca_, a process that increases the driving force for H^+^‐coupled electrogenic dipeptide absorption. This concept is depicted in Figure 8. However, other mechanisms have been suggested, such as the Ca^2+^‐dependent activation of PKC isoforms with subsequent effects of PEPT1 trafficking (Brandsch, Miyamoto, Ganapathy, & Leibach, 1994).

What is the physiological relevance of this study? Food nutrients, such as dietary dipeptides and L‐amino acids, can raise [Ca^2+^]_cyt_ in intestinal epithelial cells via CaSR activation. It is followed by activation of the basolateral IK_Ca_ channels by Ca^2+^ signaling and results in membrane hyperpolarization to promote further dipeptide absorption through a positive feedback loop. CaSR activation also triggers intracellular calcium release from the endoplasmic reticulum. Thus, CaSR‐induced Ca^2+^ signaling might sustain K^+^ channel gating to promote peptide absorption. In adults, the calcium absorption rate is 1,000 mg per day (Bronner & Pansu, 1999). Most calcium was absorbed in the jejunal via the epithelial calcium channels like TRPV6 and TRPV5 (Nijenhuis, Hoenderop, & Bindels, 2005). The absorbed calcium enters the enterocytes, some of which binds calcium‐buffering proteins and is transported inside the cell. However, not all free Ca^2+^ can bind the calcium‐buffering proteins, while some contributes refilling of ER calcium store and promotes the CaSR‐PLC‐Ca^2+^‐IK_Ca_ signaling cascade. Therefore, CaSR‐PLC‐Ca^2+^‐IK_Ca_ signaling pathway may be involved in intestinal nutrient absorption, suggesting the imbalance of this pathway may contribute to human nutritional disorders.

## CONFLICT OF INTEREST

The authors have no conflict of interest to disclose.

## AUTHOR CONTRIBUTIONS

J.X., A,Z., B.R., S.Y., O.G., H.D., B.G., E.P., U.S., and H.D. conceived and designed research; J.X., A,Z., B.R., performed experiments; J.X., B.R., and A,Z., interpreted results of experiments. J.X., A,Z., and H.D., analyzed data. J.X. and A,Z prepared figures. J.X. and H.D. drafted the manuscript. H.D. and U.S. edited and revised the manuscript. J.X., E.P., H.D., and U.S. approved the final version of the manuscript.
