# Supplementary figures and images for: Calcium‐sensing receptor regulates intestinal dipeptide absorption via Ca2+ signaling and IKCa activation
Source: Physiol Rep. 2020 Jan 21;8(1):e14337. doi: 10.14814/phy2.14337 (PMC6971415; doi:10.14814/phy2.14337)

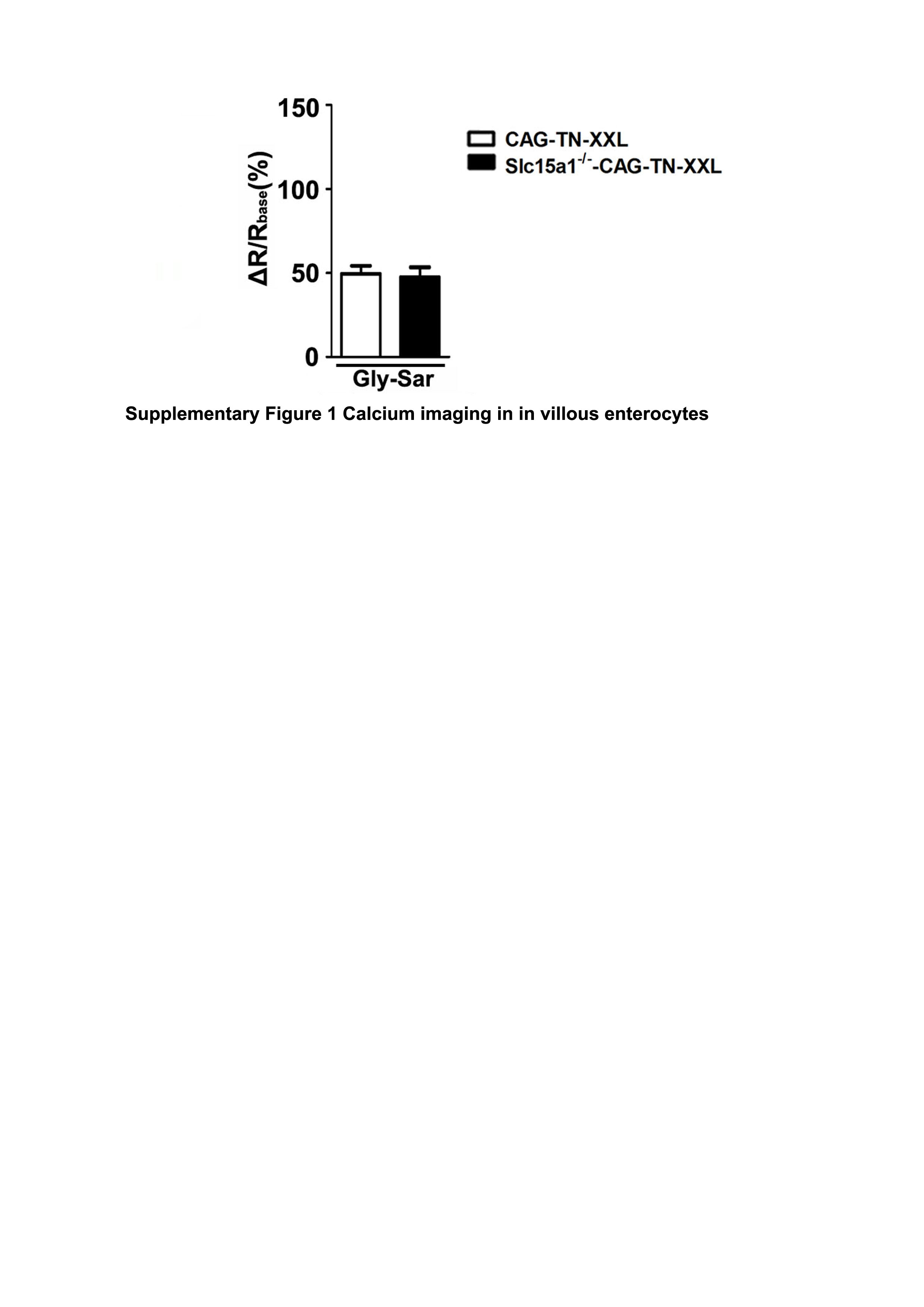

Supplement: Supplementary file 1 [file PHY2-8-e14337-s001.jpg]
